# Autophagy, apoptosis, and neurodevelopmental genes might underlie selective brain region vulnerability in attention-deficit/hyperactivity disorder

**DOI:** 10.1038/s41380-020-00974-2

**Published:** 2020-12-18

**Authors:** Jonathan L. Hess, Nevena V. Radonjić, Jameson Patak, Stephen J. Glatt, Stephen V. Faraone

**Affiliations:** 1 Department of Psychiatry, SUNY Upstate Medical University, Syracuse, NY USA; 2 Department of Neuroscience, SUNY Upstate Medical University, Syracuse, NY USA

**Keywords:** Molecular biology, Genetics

## Abstract

Large-scale brain imaging studies by the ENIGMA Consortium identified structural changes associated with attention-deficit/hyperactivity disorder (ADHD). It is not clear why some brain regions are impaired and others spared by the etiological risks for ADHD. We hypothesized that spatial variation in brain cell organization and/or pathway expression levels contribute to selective brain region vulnerability (SBRV) in ADHD. In this study, we used the largest available collection of magnetic resonance imaging (MRI) results from the ADHD ENIGMA Consortium (subcortical MRI *n* = 3242; cortical MRI *n* = 4180) along with high-resolution postmortem brain microarray data from Allen Brain Atlas (donors *n* = 6) from 22 brain regions to investigate our SBRV hypothesis. We performed deconvolution of the bulk transcriptomic data to determine abundances of neuronal and nonneuronal cells in the brain. We assessed the relationships between gene-set expression levels, cell abundance, and standardized effect sizes representing regional changes in brain sizes in cases of ADHD. Our analysis yielded significant correlations between apoptosis, autophagy, and neurodevelopment genes with smaller brain sizes in ADHD, along with associations to regional abundances of astrocytes and oligodendrocytes. The lack of enrichment of common genetic risk variants for ADHD within implicated gene sets suggests an environmental etiology to these differences. This work provides novel mechanistic clues about SBRV in ADHD.

## Introduction

Attention-deficit/hyperactivity disorder (ADHD) is a highly heritable neurodevelopmental disorder characterized by high levels of inattention, impulsivity, and hyperactivity, which frequently persist into adulthood [1]. ADHD is a relatively common disorder that affects about 5% of school-age children and 2.5% of adults [1]. Many genetic and environmental risk factors for ADHD have been documented but the mechanisms ultimately leading to the symptoms of the disorder are unknown [1].

Multi-site sMRI mega-analyses performed by the ENIGMA Consortium identified subcortical regions with significant smaller volumes in children diagnosed with ADHD compared to unaffected comparison (UC) participants, including the accumbens, amygdala, caudate, hippocampus, and putamen [2]. Stratification by age group revealed that smaller subcortical volumes were more prominent for children with ADHD than for adults with the disorder [2]. More recently, the ENIGMA Consortium found that fusiform, precentral and paracentral gyri, entorhinal cortex, and parahippocampal lobe were thinner in cases of ADHD compared to unaffected individuals spanning multiple age groups [3]. Stratifying individuals into different age groups demonstrated that children with ADHD show substantially thinner cortical regions compared to adults with ADHD. These findings reported by the ENIGMA Consortium [2, 3] join a growing body of work ascertaining that ADHD-associated deficits in regional brain volumes and cortical thickness attenuate with aging [4, 5]. It is possible that the phenomenon of selective brain region vulnerability (SBRV) may contribute to the variability of structural differences located across brain regions in ADHD.

Our conceptualization about SBRV stemmed from the theory of pathoclisis introduced in 1922 [6]. Pathoclisis posited that the basis of selective vulnerability of brain regions to risk factors of disease may be explained by variation in the constituent cell types and biochemical pathways of brain regions. Neurons are not evenly distributed across the brain, rather there is evidence that neuronal density follows an anterior–posterior gradient across the cortex [7–9]. In addition, there is evidence that gene expression in the human brain follows a distinct pattern that mirrors structural and functional organization of brain regions [10–13]. These underlying patterns related to the organization of the human brain may hold information about SBRV. We hypothesize that the organization of brain cell types and biological pathways has differential vulnerability to risk factors for ADHD, which may explain why some brain regions show structural brain changes associated with ADHD, while others do not [2, 3]. We sought to determine whether localized patterns of cell-type abundances and gene-set expression levels in brains of healthy individuals relate to the regional variation in structural brain differences seen in ADHD. Previously, we found that autophagy, apoptosis, and oxidative stress pathways were associated with smaller subcortical brain region volumes in ADHD and suggested that they may mediate SBRV [14]. The present work builds on our previous study by including newly released data from the ENIGMA-ADHD working group for cortical brain regions. We also investigate the brain cell types that may mediate SBRV in ADHD. Our goal was to determine if a distinct gene expression or distribution of cell types was characteristic of brain regions implicated in ADHD. Such data could shed light on etiological mechanisms underlying SBRV.

## Methods

**Fig. 1:**
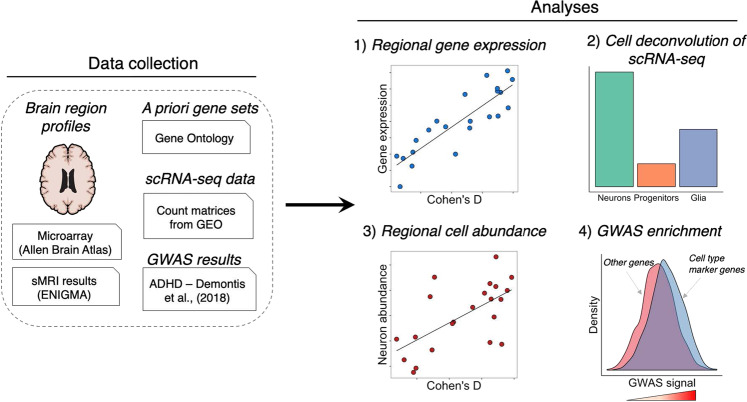
A diagram showing the general workflow used for our study. We collected regional *postmortem* brain transcriptome profiles from Allen Brain Atlas and summary statistics from two neuroimaging meta-analyses by the ENIGMA Consortium for ADHD. We analyzed the *postmortem* brain data for correlations between gene-set expression levels of apoptosis, autophagy, neurodevelopment, neurotransmitter, oxidative stress, and ADHD candidate genes with brain size differences in ADHD cases from the ENIGMA studies. We also deconvoluted the regional *postmortem* brain transcriptome profiles into discrete cell types and tested for a correlate between cell abundance and volumetric reductions in ADHD. We tested for enrichment of GWAS signals associated with ADHD among gene sets of interest (i.e., cell-type marker genes, Gene Ontology gene sets). GEO Gene Expression Omnibus, GWAS genome-wide association study, scRNA-seq single-cell RNA-sequencing.

A diagram summarizing our analytic framework is provided in Fig. 1.

### Gene sets

When identifying candidate pathways, we focused on studies that performed meta-analysis of experimental measurements among ADHD patients, or hypothesis-free pathway analysis of genome-wide signals associated with ADHD. Five biological pathways previously associated with ADHD (apoptosis, autophagy, neurotransmission, neurodevelopment, and reactive oxygen species) were selected for our current analysis [15–18]. We hypothesized that expression of these gene sets in the human brain may be associated with SBRV, which builds upon our previous study of that assessed subcortical SBRV in ADHD [14]. The latest version of the Gene Ontology database was downloaded via R package GO.db (version 3.7.0) [19] to retrieve the most up-to-date annotations for these five biological pathways: apoptosis, autophagy, neurotransmission, neurodevelopment, and reactive oxygen species [15–18]. These pathways are relatively broad and might be associated with neuropsychiatric disorders other than ADHD. A total of 1298 unique HGNC genes were identified. Precisely, 1203 of these genes had expression data in the Allen Brain Atlas. Supplementary Fig. 1 shows the number of genes contained in each gene set and the amount of pairwise overlap between gene sets.

### Structural neuroimaging data for cortical and subcortical brain regions

We used data from two international multi-site neuroimaging meta-analyses conducted by the ENIGMA-ADHD working group that evaluated structural T1-weighted brain magnetic resonance imaging (MRI) data from individuals diagnosed according to DSM-IV, with ADHD and UC participants. Summary statistics denoting case-control differences in cortical thickness and subcortical volume as standardized effect sizes (Cohen’s *d*) were obtained from published papers by the ENIGMA Consortium [2, 3]. For our primary analysis, we included results from cortical thickness measures instead of surface area. First, imaging studies have attributed changes to cortical thickness with selective vulnerability in neurodegenerative disorders, thus it is possible that cortical thickness may be sensitive to molecular cascades of SBRV in ADHD [20–23]. Second, cortical thickness and surface area both contribute to cortical volume, but cortical thickness has been shown to play a bigger role in cortical volume [24]. Therefore cortical thickness may be the better imaging phenotype to include in our analysis of the patterning of gene expression and cellular abundance across cortical and subcortical structures. For our secondary analysis, we jointly analyzed cortical thickness, cortical surface area, and subcortical volumes. Structural MRI measures had been generated using a validated MRI processing protocol with the software FreeSurfer (versions 5.1, 5.3, or 6.0). The age range for children was 4–14 years, adolescents 15–21 years, and adults 22–63 years. Differences in cortical thickness found between cases and UC participants had been adjusted for age, sex, ethnicity, and total intracranial volume (ICV), while differences in subcortical volume had been adjusted for age, sex, and total ICV. Previous ENIGMA papers showed no difference in subcortical volumes between ADHD participants who had never taken the medication compared to patients who used stimulants during their lifetime [2]. Also, no significant association was found between psychostimulant medication and cortical dimensions, neither in case-control nor in population-based designs [3]. The number of ADHD cases and UC participants included in the ENIGMA-ADHD studies is presented in Supplementary Table 1.

### Postmortem brain transcriptome profiles from adult donors

We downloaded preprocessed microarray data from the Allen Brain Atlas website (https://human.brain-map.org/) [25] containing quantile normalized transcriptome profiles for a 158 neuroanatomical substructures from five male and one female healthy donors ranging from 24 to 57 years of age (Caucasian = 3; Hispanic = 1; African American = 2). We mapped 49 of the substructures onto 22 brain regions (Supplementary Table 2) evaluated by the ENIGMA-ADHD working group (15 cortical and 7 subcortical regions), including accumbens, amygdala, caudate, cuneus, fusiform gyrus, globus pallidus, hippocampus, inferior temporal gyrus, insula, lingual gyrus, middle temporal gyrus, paracentral lobule, parahippocampal gyrus, postcentral gyrus, precentral gyrus, precuneus, putamen, superior frontal gyrus, superior parietal cortex, superior temporal gyrus, supramarginal gyrus, and thalamus. A total of 58,692 probes were used to assay transcriptome expression profiles from neuropathologically normal *postmortem* brain specimens harvested from six adult donors. Brain samples had been screened for trauma, toxicity, pathology, and history of drug or alcohol abuse, epilepsy, psychiatric or neurological disease, prion disease, infectious disease, cancer deaths, chronic renal failure, or on a ventilator for >1 h or, had a time of death > 30 h [25]. We only included microarray profiles generated on the left hemisphere for our analysis, as there were insufficient data from the right hemisphere across donors. Probe-level data were collapsed down to 19,274 HGNC genes by calculating the median log_2_ expression level of probe clusters (i.e., set of probes that measure a single gene) within each donor and brain substructure. Mean log_2_ gene expression values were then calculated among brain substructures that mapped to one of the 22 brain regions of interest evaluated by ENIGMA-ADHD. Mean log_2_ expression levels were calculated among genes annotated to each of the five a priori selected gene sets to arrive at gene-set expression levels within 22 brain regions. A plot of normalized expression values across regions of interest from Allen Brain Atlas is provided in Supplementary Fig. 2.

### Deconvolution of bulk brain tissue transcriptomes into specific brain cell types

We used a statistical cell deconvolution method from the R package dtangle [26] to infer proportional abundance of brain cell types based on expression levels of cell-type-specific marker genes from the bulk tissue transcriptome profiles from the Allen Brain Atlas. The dtangle algorithm uses a multivariate logistic function to estimate cell abundance from gene expression levels [26]. As a reference panel for deconvolution, we used single-cell RNA-sequencing (scRNA-seq) data collected from *postmortem* cortex of five male and three female healthy adult donors, ranging from 21 to 63 years of age (GEO accession ID: GSE67835) [27], which was comprised of 285 cells classified into six broad cell types, including astrocytes (*n* = 62), endothelial cells (*n* = 20), microglia (*n* = 16), neurons (*n* = 131), oligodendrocytes (*n* = 38), and oligodendrocyte progenitor cells (OPCs) (*n* = 18). We processed the scRNA-seq data by removing genes expressed at or near background levels (≤1 read counts in >90% of cells), transforming read counts for remaining genes to the log_2_ counts per million (CPM) scale with the software edgeR (v.3.24.3) [28], and quantile normalizing CPM values across cells using limma (v.3.38.3) [29]. Using dtangle, we identified cell-type-specific marker genes that were expressed above the 90th percentile in terms of fold-change in one cell type compared to all other cell types. We then deconvoluted the Allen Brain Atlas data based on observed expression of cell-type-specific markers into relative abundances of brain cell types, which can take on values between 0 and 1.

### Statistical analysis

For our primary analysis, Pearson’s correlation tests were used to determine the association between Cohen’s *d* values that reflect brain morphometric changes in ADHD cases, separated by the four case-control age groups evaluated by the ENIGMA Consortium and gene-set expression levels in “postmortem” subcortical and cortical brain regions obtained from the Allen Brain Atlas. Keeping in line with our previous study [14], the Bonferroni procedure was used to correct for multiple comparisons among association tests performed across all age bins in the imaging cohorts (children, adolescents, adults, and all participants) [30]. Based on our sample size and predefined significance threshold of *p* ≤ 0.0025 (5 gene sets × 4 age bins), we estimated having 80% power to detect a significant correlation for our primary analysis at a magnitude of Pearson’s *r* = 0.71. For our secondary analysis, we jointly analyzed all three brain imaging measurements (subcortical volume, cortical thickness, and cortical surface area) using a linear mixed model fit applying restricted maximum likelihood via the R package lmerTest (v.3.1-2). Gene-set expression was specified as a fixed-effect term, and a code for brain imaging measure was specified as a random-intercept term. Degrees of freedom and *t*-tests were computed using the Satterthwaite method. *p* values for our secondary analysis were corrected across all age groups using the Bonferroni procedure, but were treated separate from the association tests derived from our primary analysis.

A post hoc correlation test was performed to examine the relationship between Cohen’s *d* effect sizes and expression levels on a per-gene basis wherein the Bonferroni procedure was used in order to uncover genes with the strongest association with brain morphometric changes in the brains of ADHD cases among those gene sets already associated with ADHD-related brain changes (i.e., Bonferroni *p* < 0.05). Pearson’s correlation test was used to examine the relationship between abundance of specific brain cell types across subcortical and cortical brain regions along with gene-set expression levels, wherein the effective number of independent tests was the dot product of the number of independent cell types and the number of gene sets evaluated. Lastly, Pearson’s correlation tests were performed on abundance of specific brain cell types and Cohen’s *d* effect sizes for brain structural changes in ADHD separately for each case-control age group. The Bonferroni procedure was used to adjust the significance threshold and account for multiple testing across the age bins. A post hoc multivariate regression model tested whether multiple significant gene sets had conditionally independent associations with Cohen’s *d* values.

### Gene-set analysis with GWAS data for ADHD

We performed a gene-level and gene-set association analysis using the largest available GWAS meta-analysis results for ADHD with the software MAGMA (v1.07) [31, 32]. Identifiers for over 3 million single-nucleotide polymorphism (SNPs; rsIDs) and *p* values were supplied to MAGMA to perform quality control of summary statistics and compute *z* scores for gene-level associations with ADHD by averaging the observed significance values for intragenic SNPs. Gene-level associations were computed for a total of 20,274 genes, of which 1171 were included in our gene sets of interest. A genome-wide significance threshold was set at 2.47 × 10^–6^ for the gene-level tests (i.e., 2.47 × 10^−6^ = *ɑ* = $\frac{{0.05}}{{20,274}}$). With MAGMA, a linear regression model was used to perform a competitive gene-set enrichment analysis testing for differences in gene-level association scores for ADHD between a gene set and all other genes in the genome while covarying for minor allele count, gene length, number of SNPs per kilo base of the gene, and linkage disequilibrium between genes. The gene sets that we tested for GWAS enrichment included the five a priori gene sets (autophagy, apoptosis, neurodevelopment, neurotransmission regulation, and oxidative stress) and the six brain cell-type-specific marker gene lists defined in our statistical deconvolution analysis.

## Results

### Gene sets associated with smaller brain sizes in ADHD

**Fig. 2:**
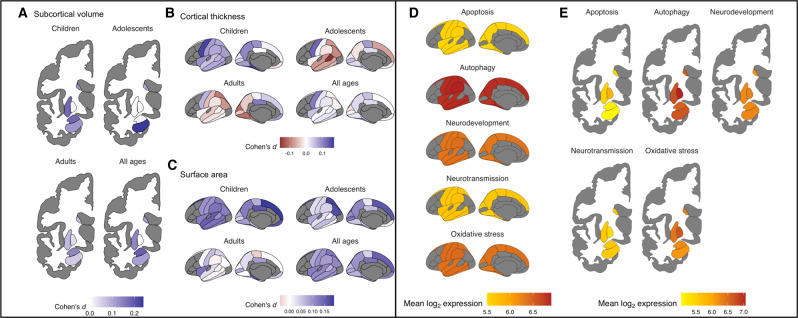
Heatmaps showing anatomical representations of neuroimaging signatures for ADHD and *postmortem* brain gene-set expression levels. **A**–**C** Standardized differences in structural MRI measurements found by the ENIGMA Consortium for ADHD are shown for subcortical volume, cortical thickness, and surface area. Larger positive values for Cohen’s *d* indicate smaller regional brain sizes among ADHD patients versus controls. **D**, **E** *Postmortem* brain gene-set expression levels for five a priori selected gene sets are presented. Expression levels for gene sets were averaged over a reference panel of six adult neurotypical donors from the Allen Brain Atlas.

**Fig. 3:**
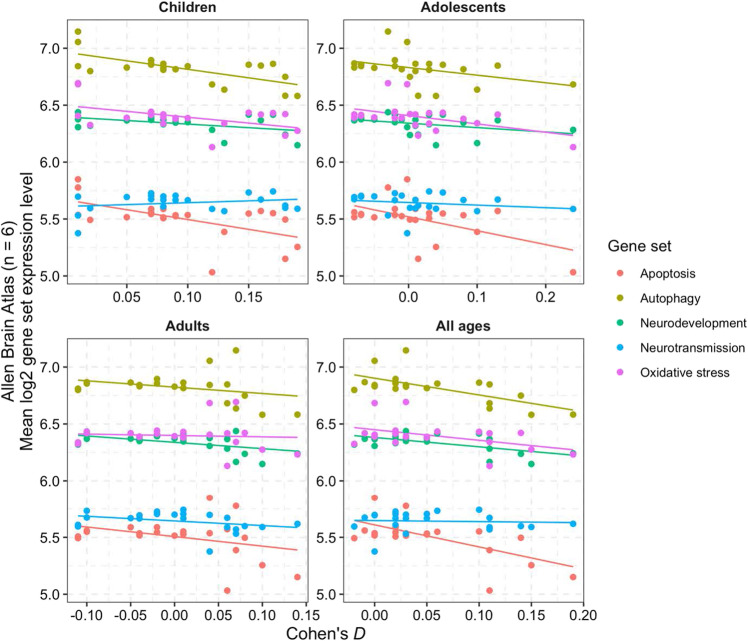
Scatterplots and best-fit regression lines show correlations between brain size differences in ADHD cases across 22 brain regions (*x*-axis, Cohen’s *d*) with gene-set expression levels (*y*-axis). Cohen’s *d* effect sizes were stratified according to the age groups described by the ENIGMA-ADHD working group in their case-control analyses of subcortical and cortical brain MRI data. Dots are color coded by gene set. Table 1 gives the statistical significance of these correlations.

Anatomical plots of the structural MRI signatures associated with ADHD alongside average expression levels of our five a priori selected gene sets are shown in Fig. 2. Figure 3 shows the correlations between the Cohen’s *d* values indexing differences between ADHD and UC participants in regional brain volumes and the gene-set expression levels for the five a priori selected gene sets. As the summary statistics for the correlation tests in Table 1 show, the expression levels of three gene sets were significantly negatively correlated with smaller brain sizes in ADHD: autophagy (children with ADHD, Pearson’s *r* = −0.67, *p* = 7.0 × 10^−4^; all ages, Pearson’s *r* = −0.72, *p* = 2.0 × 10^−4^), apoptosis (all ages, Pearson’s *r* = −0.66, *p* = 9.0 × 10^−4^), and neurodevelopment (all ages, Pearson’s *r* = −0.61, *p* = 2.4 × 10^−3^). The associations between the autophagy and apoptosis gene sets with smaller brain sizes disappeared when the two gene sets were both specified as predictors in a post hoc multiple linear regression model that used Cohen’s *d* values from ENGIMA’s analysis of all ADHD cases and UC participants as the outcome variable (*p* values = 0.072 and 0.76, respectively), suggesting that these gene sets are not contributing conditionally independent effects on brain size differences in ADHD. This finding is consistent with the fact that the autophagy and apoptosis gene sets share a significantly greater number of genes than expected by chance (Supplementary Fig. 1). Our joint analysis of cortical thickness, cortical surface area, and subcortical volume data added further support to the associations for autophagy and apoptosis gene sets (Supplementary Table 3). The neurodevelopment gene set was not significant in the joint linear mixed model. However, the neurotransmission gene set became significant for two age groups after Bonferroni correction: children with ADHD (standardized *β* = 0.72, SE = 0.19, df = 34.6, *p* = 5.8 × 10–4) and all ages (standardized *β* = 0.69, SE = 0.19, df = 33.52, *p* = 0.0074).

**Table 1:** Correlation of gene-set expression levels with brain size differences in ADHD cases across 22 brain regions.

	Children		Adolescents		Adults		All ages	
Gene set	Pearson’s *r* [95% CI]	*p* value	Pearson’s *r* [95% CI]	*p* value	Pearson’s *r* [95% CI]	*p* value	Pearson’s *r* [95% CI]	*p* value
Apoptosis	−0.53 [−0.78 to −0.14]	0.01	−0.53 [−0.78 to −0.13]	0.01	−0.4 [−0.7 to 0.03]	0.07	**−0.66 [−0.84** to **−0.33]**	**0.0009**
Autophagy	**−0.67 [−0.85** to**−0.34]**	**0.0007**	−0.4 [−0.71 to 0.02]	0.06	−0.4 [−0.7 to 0.03]	0.07	**−0.72 [−0.88** to **−0.43]**	**0.0002**
Neurodevelopment	−0.39 [−0.7 to 0.03]	0.07	−0.36 [−0.68 to 0.08]	0.1	−0.59 [−0.81 to −0.23]	0.0038	**−0.61 [−0.82** to **−0.26]**	**0.0024**
Neurotransmission	0.21 [−0.23 to 0.58]	0.34	−0.21 [−0.58 to 0.23]	0.34	−0.34 [−0.67 to 0.09]	0.12	−0.07 [−0.48 to 0.36]	0.76
Oxidative stress	−0.48 [−0.75 to −0.07]	0.02	−0.48 [−0.75 to −0.07]	0.02	−0.12 [−0.51 to 0.32]	0.61	−0.47 [−0.75 to −0.07]	0.03

Associations in boldface showed a corrected *p* value < 0.05 after applying the Bonferroni procedure across all correlation tests.

*CI* confidence interval.

Among 684 genes tested, 27 showed a significant association with brain size differences in ADHD at a Bonferroni *p* < 0.05 (Supplementary Fig. 3). Expression levels of TAO kinase 2 (*TAOK2*), a serine/threonine kinase gene, had the most significant association with smaller brain volumes in ADHD (Pearson’s *r* = −0.86, uncorrected *p* = 2.8 × 10^−7^, Bonferroni *p* = 3.8 × 10^−4^). A visualization of the developmental trajectory of *TAOK2* gene expression in *postmortem* human brain tissue from 8 postconception weeks (pcw) to 40 years of age is provided in Supplementary Fig. 4. *TAOK2* expression levels are shown to be lowest at 8 pcw. Expression levels rise during prenatal development reaching a peak at 25 pcw, followed by a moderate decline until 4 years old and a slight increase through adulthood.

### Association of cell-type abundance with gene sets and volumetric changes in ADHD

**Fig. 4:**
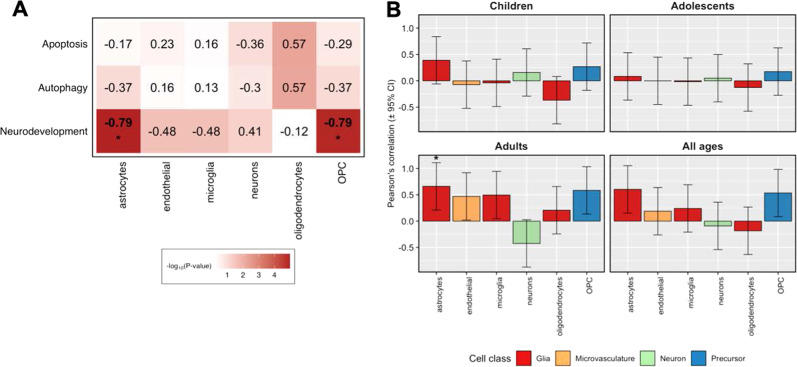
Brain cell abundances show correlations with gene-set expression levels and brain size differences seen in ADHD. **A** Correlation of gene-set expression levels from 22 brain regions with abundances of brain cells estimated through deconvolution of transcriptome profiles from bulk tissue samples of adult *postmortem* brain samples in Allen Brain Atlas. This analysis was limited to gene sets that showed a significant association from our primary analysis that examined the correlation between gene-set expression levels and brain size changes associated with ADHD. **B** Estimates of neuronal and nonneuronal cell abundance based on deconvolution of Allen Brain Atlas guided by scRNA-seq data from adult cortex samples show correlations with brain size differences in ADHD. Associations marked by asterisks (*) reached a Bonferroni-corrected significance level of *p* < 0.05. OPC oligodendrocyte progenitor cells.

Figure 4A shows significant associations found between four brain cell types and autophagy, apoptosis, neurodevelopment, or oxidative stress genes (Bonferroni *p* < 0.05). The cell types implicated by these associations were: astrocytes, neurons, oligodendrocytes, and OPCs. Brain size differences in ADHD were significantly associated with the abundance of astrocytes and OPCs (Bonferroni *p* < 0.05, Fig. 4B).

### Association of genes and gene sets with ADHD from GWAS meta-analysis

Genome-wide significant gene-level associations with ADHD were found for three genes via gene-based association test with MAGMA, one of which belongs to the autophagy gene set (*KDM4A*, *p* = 2.61 × 10^−11^). The other two genes are members of the neurotransmission gene set (*CUBN*, *p* = 5.2 × 10^−7^ and *MEF2C*, *p* = 6.33 × 10^−7^). Competitive gene-set enrichment did not uncover a significant association between autophagy, apoptosis, neurodevelopment, neurotransmission, or oxidative stress gene sets and ADHD (Supplementary Table 4). Brain cell-type-specific marker gene lists did not show enrichment of GWAS signals for ADHD (Supplementary Table 4).

## Discussion

This work sought to better understand SBRV in ADHD. It extends our prior study of subcortical regions [14] by adding cortical data and by identifying the cell types possibly contributing to our results. Although we must be cautious in interpreting our results, they suggest that SBRV in ADHD may be due to temporo-spatial variation in the expression levels of autophagy and apoptosis genes. In adulthood, neurodevelopmental genes also appear to play a role in SBRV.

We previously reported a significant association between smaller subcortical brain volumes and spatial variation in expression of oxidative stress genes [14]. However, after having nearly tripled our sample size, the association between oxidative stress genes and SBRV in ADHD was markedly diminished. Conversely, the significance of the associations between Cohen’s *d* and autophagy genes and apoptosis genes became stronger in this study compared to our previous findings [14], supporting the idea that the autophagy and apoptosis pathways (which share expressed genes) mediate SBRV for both cortical and subcortical structures. Although not parsimonious, it is plausible that neuronal or nonneuronal cells within cortical regions are more tolerant to oxidative stress compared to brain cells in subcortical regions and that oxidative stress may play a role only for subcortical structures.

Compared with our previous work, the association between SBRV in ADHD and the neurodevelopment gene set is a novel finding. Neurodevelopmental genes play a critical role in the proliferation and differentiation of progenitor cells and the formation of neural circuits during fetal life and infancy. In adulthood, neurodevelopmental genes also play an important role in synaptic and dendritic development, myelination and adult neurogenesis [33]. Thirty nine of the 263 genes (hypergeometric enrichment *p* value = 2.5 × 10^−29^) in the neurodevelopmental gene set showed evidence of regulating adult neurogenesis based on the Mammalian Adult Neurogenesis Gene Ontology database [34]. The neurodevelopmental gene set did not remain significantly associated with brain size differences associated with ADHD when analyzed in our joint linear mixed model, which is possibly due to the ontogenetic differences between cortical thickness and cortical surface area. Nevertheless, further examination into the role of neurodevelopmental genes on SBRV in ADHD is warranted based on the result from our primary analysis and evidence from the literature. Support for a role played by neurodevelopmental genes in ADHD was also reported by Poelmans et al.’s [18] analysis of GWAS data. According to our cell deconvolution analysis, brain regions showing smaller volumes in ADHD during development also showed elevated numbers of astrocytes and OPCs in adulthood. These results imply that compensation for volumetric loss seen in adulthood could be attributed not only to increases in the number of neurons, but also to a compensatory response of glial cells. Indeed, animal models of ADHD have shown increased number of GFAP (astrocyte-specific marker) positive cells in the spontaneous hypertensive rat model [35] and astrocytosis in thalamus and cortex of Git1^−/−^ mice [36]. Importantly, white matter abnormalities have been reported in ADHD [37]. Oligodendrocytes are the main myelinating cells in the central nervous system [38]. Myelination accounts for ~40% of human brain parenchyma and could be an important contributor to the differences in brain volume [39]. Numerous reports link autophagy, cell survival pathways, and upstream signaling cascades, such as PI3K-mTOR, in the regulation of myelination [40]. Furthermore, one of the top GWAS signals for ADHD is located on chromosome 1 in *ST3GAL3*, a sialyltransferase gene linked with intellectual disability [41], which has also been linked with cognitive deficits and demyelination in genetically modified mice deficient in St3gal3 [42]. Additional genetic, neuroimaging, and experimental studies are warranted to refine our understanding about mechanisms of myelination in relation to brain volume differences in ADHD.

Taken together, we hypothesize that the upregulation of neurodevelopment genes during adulthood might compensate for reduced brain volumes early in life. Although we need mechanistic studies to fully explain why the structural brain deficits found in ADHD in youth attenuate in adulthood [2, 3, 43] and to define the biological substrate for the brain maturation delay theory of ADHD [4], some clues are provided by existing genetic epidemiologic data. A review of twin studies of ADHD show that, after accounting for rater effects, the heritability of ADHD is stable across the lifespan [44]. Mathematical modeling of twin data shows that different gene sets are active at different stages of development. This latter finding is consistent with the finding that while the genetic correlation between childhood and adult GWAS results is high (0.81), it is imperfect [45]. These findings suggest that the gene sets involved in the onset and persistence of ADHD are not isomorphic. Future studies should attempt to isolate those gene sets accounting for persistence. We hypothesize that they will overlap with those implicated in the present work.

Expression of the gene encoding *TAOK2* had the strongest correlation with SBRV for adults with ADHD. Spatial expression profiles taken from the latest RNA-sequencing data from Genotype-Tissue Expression version 7 [46] show that *TAOK2* is widely expressed in the central nervous system, especially the cerebellum. *TAOK2* is part of the neurodevelopment gene set and has been shown to regulate brain size and neural connectivity, and is present in the 16p11.2 microdeletion region, a rare structural variant that has been associated with range of neurodevelopmental phenotypes including autism, ADHD, and intellectual disability [47–51]. Deletion of *Taok2* in mice was found to lead to deficits in dendritic growth, synaptic formation, cortical layering, and autism-associated phenotypes [51].

*TAOK2* expression levels typically peak during prenatal brain development, but levels remain fairly consistent through development and maturation (Supplementary Fig. 4). We postulate that the upregulation of *TAOK2* affects brain development and ultimately results in smaller brain regions seen in ADHD, but *postmortem* brain data from ADHD patients are needed to confirm this hypothesis.

There is indirect support for the hypothesis that SBRV in ADHD may have an environmental etiology. We found that none of the biological pathways we identified as associated with SBRV were associated with ADHD in gene-set analyses of a very large GWAS of ADHD. The 27 genes that were significantly correlated with SBRV in ADHD were not significantly associated with ADHD as a gene set based on the most current GWAS meta-analysis GWAS of ADHD [32]. It is possible that our chosen gene sets could be significantly impacted by *trans*-acting genetic variants related to ADHD, but those signals are not detected by the gene-set enrichment analysis approach used in our study. The gene sets chosen for our current study could be refined through gene expression analysis in ADHD patients, which increases the likelihood of uncovering genes that are impacted by DNA variants related to ADHD. Consistent with the idea that SBRV may have a primarily environmental etiology, another study used linkage disequilibrium score regression (LDSC) to show that, although ADHD was genetically correlated with ICV (*r*_*g*_ = −0.23, *p* = 1.5 × 10^−4^), it was not genetically correlated with subcortical brain structure volumes corrected for ICV [52]. Those findings indicate little or no overlap between the common genetic variants that cause ADHD and those that regulate subcortical SBRV. In contrast to these findings, Grasby et al. [53] found that ADHD showed significant genetic correlations with variation in cortical thickness of the inferior parietal (*r*_*g*_ = 0.22, SE = 0.069, *p* = 0.001), lingual cortex (*r*_*g*_ = 0.19, SE = 0.093, *p* = 0.039), and fusiform gyrus (*r*_*g*_ = −0.23, SE = 0.11, *p* = 0.033) based on LDSC, suggesting that common risk variants could play a small role in cortical SBRV in ADHD. The literature on rare variants in ADHD is too sparse [54, 55] to assess what role they might play in the disorder.

Given these weak genetic findings, we hypothesize that environmental risks and their interactions with genes may explain SBRV in ADHD, although this idea should be viewed with caution until additional supportive data are available. Given the observational nature of the ENIGMA studies, we cannot firmly conclude that structural MRI differences seen in ADHD are causal of the disorder, or occur as a consequence of ADHD. A possible consequential effect on brain size differences in medication use. Although medication use was accounted for in the ENIGMA studies, Hoogman et al. [3] found that psychostimulant use was nominally significant associated with smaller surface area among children with ADHD, suggesting that medications may explain some of the brain size differences seen in ADHD. In contrast, Hoogman et al. [3] found through familial analysis that non-ADHD siblings had similar structural MRI characteristics as their ADHD-affected siblings, demonstrating that ADHD diagnosis was not necessary for brain size differences to be observed, implying that shared genetic or environmental factors play an intimate role in brain size differences seen in ADHD. Alternatively, it is possible that weak genetic correlations of ADHD with structural brain phenotypes suggest that complex genetic relationships mediate SBRV. This led us to propose the following theoretical model of SBRV in ADHD which currently promotes an environmental explanation, but leaves open the potential role of genetic factors (illustrated in Supplementary Fig. 5). Our model attributes the initial etiology of SBRV to environmental events that threaten neural integrity. Examples of relevant environmental risks known to be associated with ADHD are pregnancy and delivery complications, low birth weight, and maternal alcohol consumption during pregnancy [56]. In our model, these events lead to a cascade of events that cause: (a) stressed neurons and astrocytes, (b) dysregulated autophagy and apoptosis pathways, and (c) cell death. SBRV occurs because some brain regions are better protected from this cascade due to the degree to which they express genes in pathways that regulate apoptosis and autophagy. Those regions that are weakly protected suffer more cell loss than other regions leading to SBRV. Some cases of ADHD and ADHD-associated volumetric reductions persist into adulthood [2, 4, 43, 57]. Our results from the adult ADHD analyses suggest that the association of the neurodevelopmental gene set with SBRV in adult ADHD may account for this persistence. Because the neurodevelopmental gene set is involved in neurogenesis, synaptic and dendritic development, and myelination in adolescence and adulthood, we postulate that those brain regions that express these pathways to a greater degree are more likely than other brain regions to recover from SBRV.

There are limitations in our study that must be considered when interpreting our results. Our work is motivated by our theory of SBRV in ADHD, which is based on many assumptions as described above. If our results are not robust to violations of these assumptions our conclusion would be called into questions. We cannot quantify this robustness but can point out weaknesses as follows. We could only provide indirect evidence of pathways potentially relevant to SBRV in ADHD, as causal inferences could not be drawn from the cross-sectional data used in our study. Ideally, studies of gene expression in brain tissue from ADHD patients could be used to confirm our results but no such tissue resources exist. The use of neurotypical samples from the Allen Brain Atlas is informative because it allows us to infer biological pathways that might be affected by the DNA variants associated with ADHD. The Allen Brain Atlas *postmortem* brain transcriptome data were based on a relatively small sample of normal adult brains; thus, our results might not generalize to all populations. Additionally, as the gene expression data and brain volume data are not from the same sample, it is difficult to infer whether pathways are upregulated or downregulated. The small sample size was prohibitive for assessing sex-specific profiles of brain gene expression; however, sex-related differences are unlikely to explain our findings since ENIGMA showed that brain volumetric changes associated with ADHD were not different between males and females. The use of summary statistics from ENIGMA limited the ability to assess for the familial effects; however, two subsets of ENIGMA-ADHD samples show no difference in cortical regions and level of ADHD symptoms in unaffected siblings compared to the controls. Interhemispheric differences could not be accounted for since Allen Brain Atlas assessed a single (left) hemisphere, and the ENIGMA-ADHD working group averaged volumetric differences in ADHD across right and left hemispheres. Different brain structure nomenclatures were used by the Allen Brain Atlas (neuroanatomical ontology) and ENIGMA-ADHD (Desikan–Killian Atlas for cortical parcellation), which could have potentially added unwanted variation to our analysis.

Despite these limitations, our work is beginning to clarify potential causes of SBRV in ADHD and to provide hypotheses for testing in future research. By identifying specific cell types and biological pathways affected in ADHD, we provide guidance for future in vitro studies to test our theoretical model. Such studies may uncover genetic or environmental factors that influence SBRV and lead to cellular models that can be used to develop new medications for the disorder.

## Supplementary information

Supplemental Materials
