# Supplementary material for: Autophagy, apoptosis, and neurodevelopmental genes might underlie selective brain region vulnerability in attention-deficit/hyperactivity disorder
Source: Mol Psychiatry. 2020 Dec 18;26(11):6643–54. doi: 10.1038/s41380-020-00974-2 (PMC8760041; doi:10.1038/s41380-020-00974-2)
Supplement: Supplementary file 1 — Supplemental Materials [file 41380_2020_974_MOESM1_ESM.docx]

**Supplementary Materials**

**Authors**: Jonathan L. Hess^1^, Nevena V. Radonjić^1^, Jameson Patak^2^, Stephen J. Glatt^1^, and Stephen V. Faraone^1^

**Affiliations:**

^1^ Department of Psychiatry, SUNY Upstate Medical University; Syracuse, NY, USA

^2^ Department of Neuroscience, SUNY Upstate Medical University; Syracuse, NY, USA

Table of Contents

[Supplementary Tables 2](#_Toc49161885)

[Supplementary Table 1. 2](#_Toc49161886)

[Supplementary Table 2. 3](#_Toc49161887)

[Supplementary Table 3. 4](#_Toc49161888)

[Supplementary Table 4. 5](#_Toc49161889)

[Supplementary Figures 6](#_Toc49161890)

[Supplementary Figure 1 6](#_Toc49161891)

[Supplementary Figure 2 7](#_Toc49161892)

[Supplementary Figure 3 8](#_Toc49161893)

[Supplementary Figure 4 9](#_Toc49161894)

[Supplementary Figure 5 10](#_Toc49161895)

[References 11](#_Toc49161896)

# Supplementary Tables

Supplementary Table 1. Sample sizes from the sMRI studies of ADHD performed by the ENIGMA-ADHD working group.

| **sMRI cohorts** | **Children** | | | **Adolescents** | | | **Adults** | | | **All** | | |
| --- | --- | --- | --- | --- | --- | --- | --- | --- | --- | --- | --- | --- |
| **Subcortical** ^1^ | N cases | N controls | N total | N cases | N controls | N total | N cases | N controls | N total | N cases | N controls | N total |
| Accumbens | 810 | 827 | 1,637 | 323 | 224 | 547 | 510 | 415 | 925 | 1,652 | 1,471 | 3,123 |
| Amygdala | 767 | 820 | 1,587 | 321 | 226 | 547 | 500 | 412 | 912 | 1,598 | 1,463 | 3,061 |
| Caudate | 825 | 840 | 1,665 | 324 | 224 | 548 | 502 | 420 | 922 | 1,659 | 1,489 | 3,148 |
| Hippocampus | 764 | 802 | 1,566 | 320 | 225 | 545 | 506 | 404 | 910 | 1,599 | 1,436 | 3,035 |
| Pallidum | 816 | 831 | 1,647 | 321 | 223 | 544 | 506 | 412 | 918 | 1,651 | 1,471 | 3,122 |
| Putamen | 836 | 854 | 1,690 | 329 | 228 | 557 | 499 | 416 | 915 | 1,660 | 1,497 | 3,157 |
| Thalamus | 604 | 616 | 1,220 | 288 | 202 | 490 | 503 | 416 | 919 | 1,405 | 1,242 | 2,647 |
| **Cortical thickness** ^2^ |  |  |  |  |  |  |  |  |  |  |  |  |
| Cuneus | 1,076 | 1,047 | 2,123 | 432 | 346 | 778 | 732 | 539 | 1,271 | 2,240 | 1,932 | 4,172 |
| Fusiform | 1,077 | 1,044 | 2,121 | 428 | 345 | 773 | 687 | 493 | 1,180 | 2,192 | 1,882 | 4,074 |
| Inferior temporal | 1,065 | 1,040 | 2,105 | 408 | 336 | 744 | 683 | 493 | 1,176 | 2,156 | 1,869 | 4,025 |
| Insula | 1,079 | 1,043 | 2,122 | 428 | 344 | 772 | 725 | 532 | 1,257 | 2,232 | 1,919 | 4,151 |
| Lingual | 1,081 | 1,046 | 2,127 | 429 | 344 | 773 | 688 | 494 | 1,182 | 2,198 | 1,884 | 4,082 |
| Middle temporal | 1,025 | 1,001 | 2,026 | 389 | 323 | 712 | 670 | 477 | 1,147 | 2,084 | 1,801 | 3,885 |
| Paracentral | 1,075 | 1,047 | 2,122 | 431 | 347 | 778 | 732 | 538 | 1,270 | 2,238 | 1,932 | 4,170 |
| Parahippocampal | 1,076 | 1,041 | 2,117 | 429 | 345 | 774 | 688 | 492 | 1,180 | 2,193 | 1,878 | 4,071 |
| Postcentral | 1,059 | 1,034 | 2,093 | 427 | 345 | 772 | 727 | 528 | 1,255 | 2,213 | 1,907 | 4,120 |
| Precentral | 1,064 | 1,040 | 2,104 | 425 | 344 | 769 | 729 | 537 | 1,266 | 2,218 | 1,921 | 4,139 |
| Precuneus | 1,080 | 1,044 | 2,124 | 431 | 347 | 778 | 732 | 539 | 1,271 | 2,243 | 1,930 | 4,173 |
| Superior frontal | 1,074 | 1,044 | 2,118 | 431 | 347 | 778 | 730 | 535 | 1,265 | 2,235 | 1,926 | 4,161 |
| Superior parietal | 1,073 | 1,045 | 2,118 | 430 | 347 | 777 | 730 | 539 | 1,269 | 2,233 | 1,931 | 4,164 |
| Superior temporal | 995 | 990 | 1,985 | 378 | 319 | 697 | 661 | 476 | 1,137 | 2,034 | 1,785 | 3,819 |
| Supramarginal | 1,064 | 1,039 | 2,103 | 426 | 343 | 769 | 728 | 535 | 1,263 | 2,218 | 1,917 | 4,135 |

Note: The age range for children was 4 – 14 years, adolescents 15 – 21, and adults 22 – 63 years

Supplementary Table 2. Identities of brain structures assessed by ENIGMA-ADHD that were represented in the Allen Brain Atlas.

| **ENIGMA region label** | **Allen Brain Atlas substructure name** |
| --- | --- |
| accumbens | nucleus accumbens, left |
| amygdala | amygdalohippocampal transition zone, left |
| caudate | body of caudate nucleus, left |
| caudate | head of caudate nucleus, left |
| caudate | tail of caudate nucleus, left |
| cuneus | cuneus, left, peristriate |
| cuneus | cuneus, left, striate |
| fusiform | fusiform gyrus, left, bank of cos |
| fusiform | fusiform gyrus, left, bank of the its |
| globus pallidus | globus pallidus, external segment, left |
| globus pallidus | globus pallidus, internal segment, left |
| hippocampus | dentate gyrus, left |
| hippocampus | CA1 field, left |
| hippocampus | CA2 field, left |
| hippocampus | CA3 field, left |
| hippocampus | subiculum, left |
| inferior temporal gyrus | inferior temporal gyrus, left, bank of mts |
| inferior temporal gyrus | inferior temporal gyrus, left, bank of the its |
| inferior temporal gyrus | inferior temporal gyrus, left, lateral bank of gyrus |
| insula | long insular gyri, left |
| insula | short insular gyri, left |
| lingual cortex | lingual gyrus, left, peristriate |
| lingual cortex | lingual gyrus, left, striate |
| middle temporal gyrus | middle temporal gyrus, left, inferior bank of gyrus |
| middle temporal gyrus | middle temporal gyrus, left, superior bank of gyrus |
| paracentral gyrus | paracentral lobule, anterior part, left |
| paracentral gyrus | paracentral lobule, anterior part, left, inferior bank of gyrus |
| parahippocampal gyrus | parahippocampal gyrus, left, bank of the cos |
| parahippocampal gyrus | parahippocampal gyrus, left, lateral bank of gyrus |
| postcentral gyrus | postcentral gyrus, left, bank of the central sulcus |
| postcentral gyrus | postcentral gyrus, left, bank of the posterior central sulcus |
| postcentral gyrus | postcentral gyrus, left, inferior lateral aspect of gyrus |
| postcentral gyrus | postcentral gyrus, left, superior lateral aspect of gyrus |
| precentral gyrus | precentral gyrus, left, bank of the central sulcus |
| precentral gyrus | precentral gyrus, left, bank of the precentral sulcus |
| precentral gyrus | precentral gyrus, left, inferior lateral aspect of gyrus |
| precentral gyrus | precentral gyrus, left, superior lateral aspect of gyrus |
| precuneus | precuneus, left, inferior lateral bank of gyrus |
| precuneus | precuneus, left, superior lateral bank of gyrus |
| putamen | putamen, left |
| superior frontal gyrus | superior frontal gyrus, left, lateral bank of gyrus |
| superior frontal gyrus | superior frontal gyrus, left, medial bank of gyrus |
| superior parietal lobule | superior parietal lobule, left, inferior bank of gyrus |
| superior parietal lobule | superior parietal lobule, left, superior bank of gyrus |
| superior temporal gyrus | superior temporal gyrus, left, inferior bank of gyrus |
| superior temporal gyrus | superior temporal gyrus, left, lateral bank of gyrus |
| supramarginal gyrus | supramarginal gyrus, left, inferior bank of gyrus |
| supramarginal gyrus | supramarginal gyrus, left, superior bank of gyrus |
| thalamus | reticular nucleus of thalamus, left |

Supplementary Table 3. Joint analysis of subcortical volumes, cortical thickness, and cortical surface area with linear mixed models show associations between regional gene set expression levels in *postmortem* brain and regional brain size differences attributed to ADHD. Results shown in **bold** survived correction with the Bonferroni procedure across all association tests. Note: standardized beta coefficients are shown.

|  | **Children** | | | **Adolescents** | | | **Adults** | | | **All ages** | | |
| --- | --- | --- | --- | --- | --- | --- | --- | --- | --- | --- | --- | --- |
| **Gene set** | Beta | SE | *p*-value | Beta | SE | *p*-value | Beta | SE | *p*-value | Beta | SE | *p*-value |
| Apoptosis | -0.46 | 0.15 | 4.20E-03 | -0.37 | 0.15 | 0.02 | -0.11 | 0.14 | 0.45 | **-0.43** | **0.12** | **1.23E-03** |
| Autophagy | **-0.54** | **0.14** | **5.64E-04** | -0.25 | 0.15 | 0.11 | -0.12 | 0.13 | 0.39 | **-0.45** | **0.11** | **3.69E-04** |
| Neurodevelopment | -0.32 | 0.17 | 0.08 | -0.19 | 0.19 | 0.31 | 0.04 | 0.17 | 0.80 | -0.26 | 0.17 | 0.13 |
| Neurotransmission | **0.72** | **0.19** | **5.82E-04** | 0.01 | 0.19 | 0.94 | 0.43 | 0.17 | 0.02 | **0.69** | **0.15** | **3.78E-05** |
| Oxidative stress | -0.40 | 0.15 | 0.01 | -0.36 | 0.14 | 0.01 | -0.02 | 0.13 | 0.88 | -0.34 | 0.12 | 7.49E-03 |

Supplementary Table 4. Gene set association analysis with data from the largest available GWAS meta-analysis of ADHD published by Demontis *et al*., (2018).

| **Source of gene set** | **Gene set** | **# of genes** | **Beta** | **SE** | ***p*-value** |
| --- | --- | --- | --- | --- | --- |
| Gene Ontology | Apoptosis | 35 | -0.05 | 0.16 | 0.615 |
|  | Autophagy | 353 | 0.03 | 0.05 | 0.237 |
|  | Neurodevelopment | 243 | 0.04 | 0.06 | 0.254 |
|  | Neurotransmission | 335 | 0.06 | 0.05 | 0.122 |
|  | Oxidative stress | 302 | 0.01 | 0.05 | 0.417 |
| Adult cortex scRNA-seq | OPC | 122 | 0.04 | 0.09 | 0.323 |
|  | astrocytes | 170 | -0.05 | 0.07 | 0.775 |
|  | endothelial | 166 | -0.09 | 0.08 | 0.887 |
|  | microglia | 180 | -0.01 | 0.07 | 0.545 |
|  | neurons | 184 | 0.04 | 0.07 | 0.272 |
|  | oligodendrocytes | 126 | -0.11 | 0.08 | 0.914 |

# Supplementary Figures


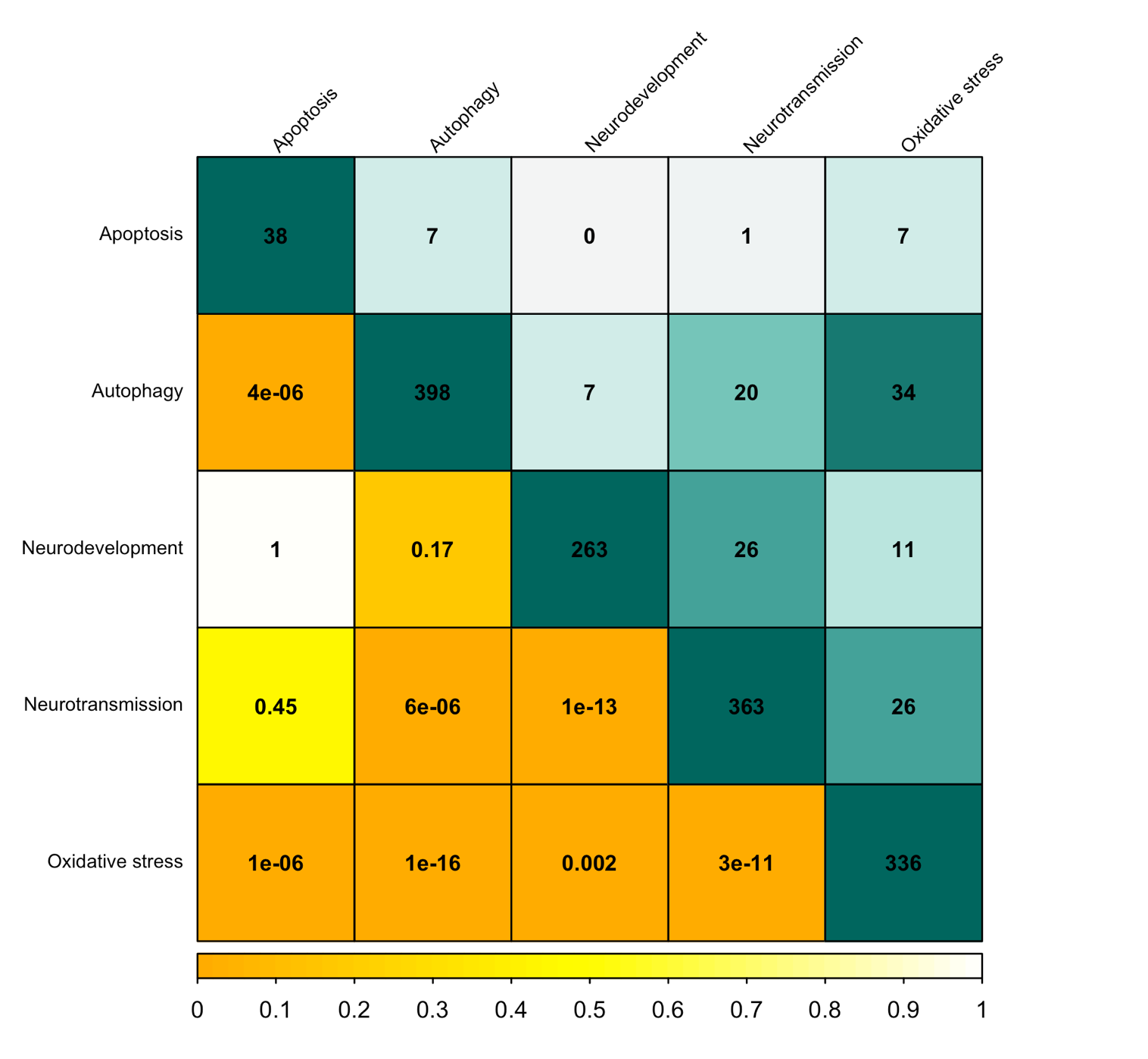


Supplementary Figure 1. The total number of genes within each gene set (along diagonal) along with the number of shared genes identified between pairs of gene sets (above diagonal). One-tailed Fisher’s *p*-values are provided below the diagonal, indicating whether the number of shared genes identified between pairs of gene sets was greater than expected by chance.


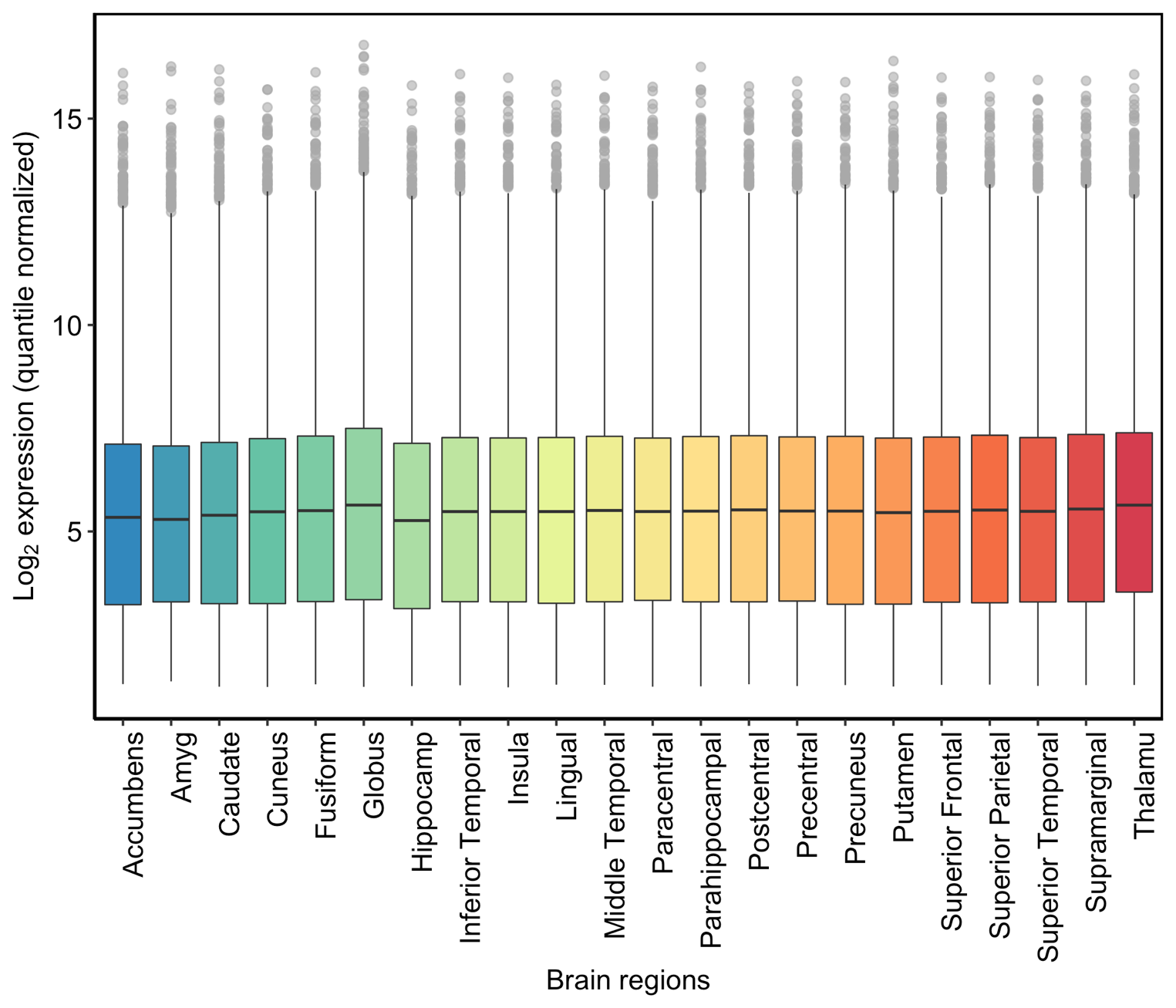


Supplementary Figure 2. Log_2_ quantile-normalized transcriptome profiles of 22 brain regions using data obtained from the Allen Brain Atlas ^3^.


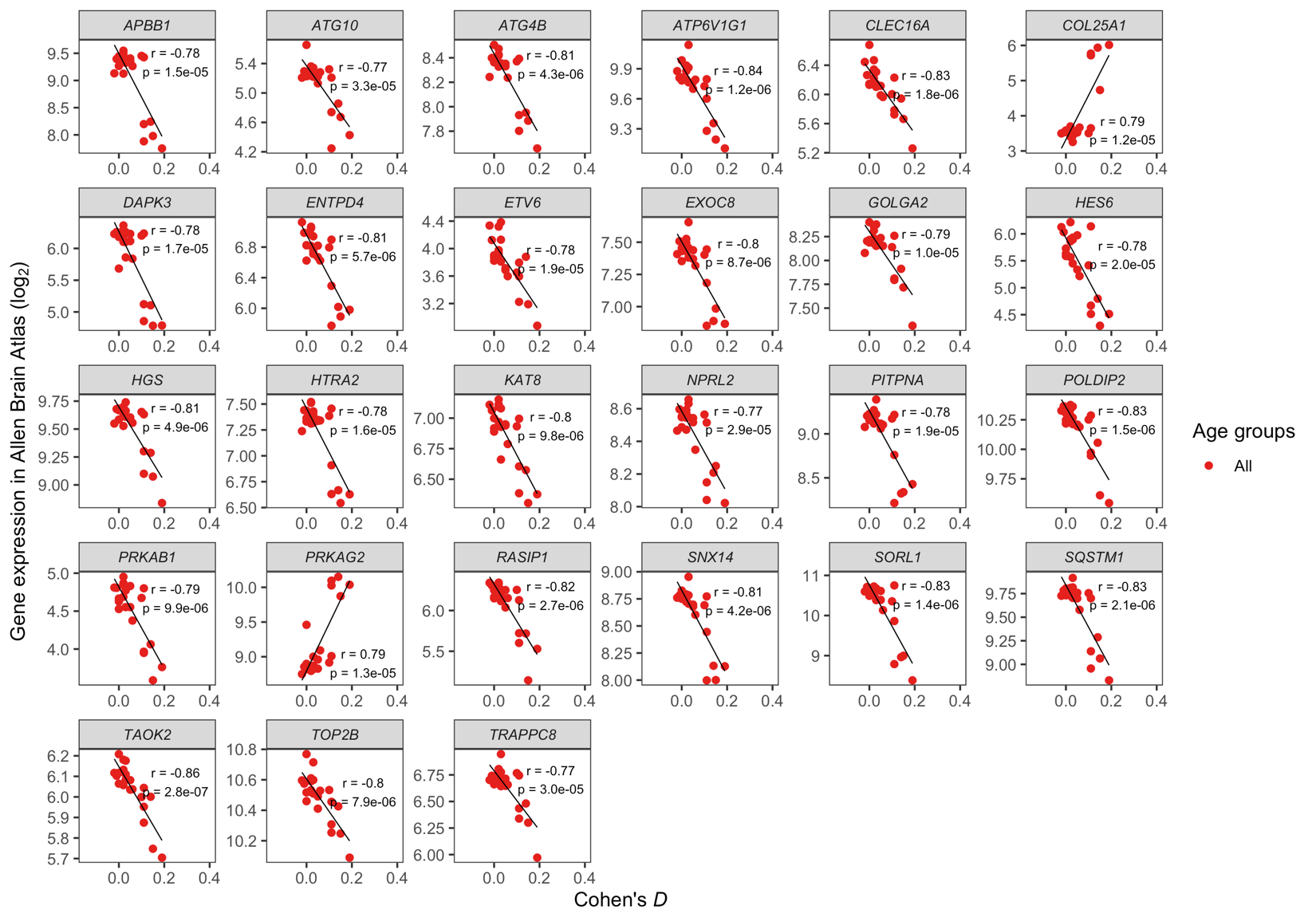


Supplementary Figure 3. Scatterplots depicting the significant correlations between gene expression levels for 27 genes and brain size differences in ADHD cases (larger value of Cohen’s *d* refers to smaller brain sizes in ADHD). These associations had a Bonferroni-adjusted *p* < 0.05 after correcting for 974 genes tested within each of the four case-control age groups evaluated by the ENIGMA-ADHD working group. A best-fit regression line and shaded band denoting the standard error of the correlation is provided in each panel.


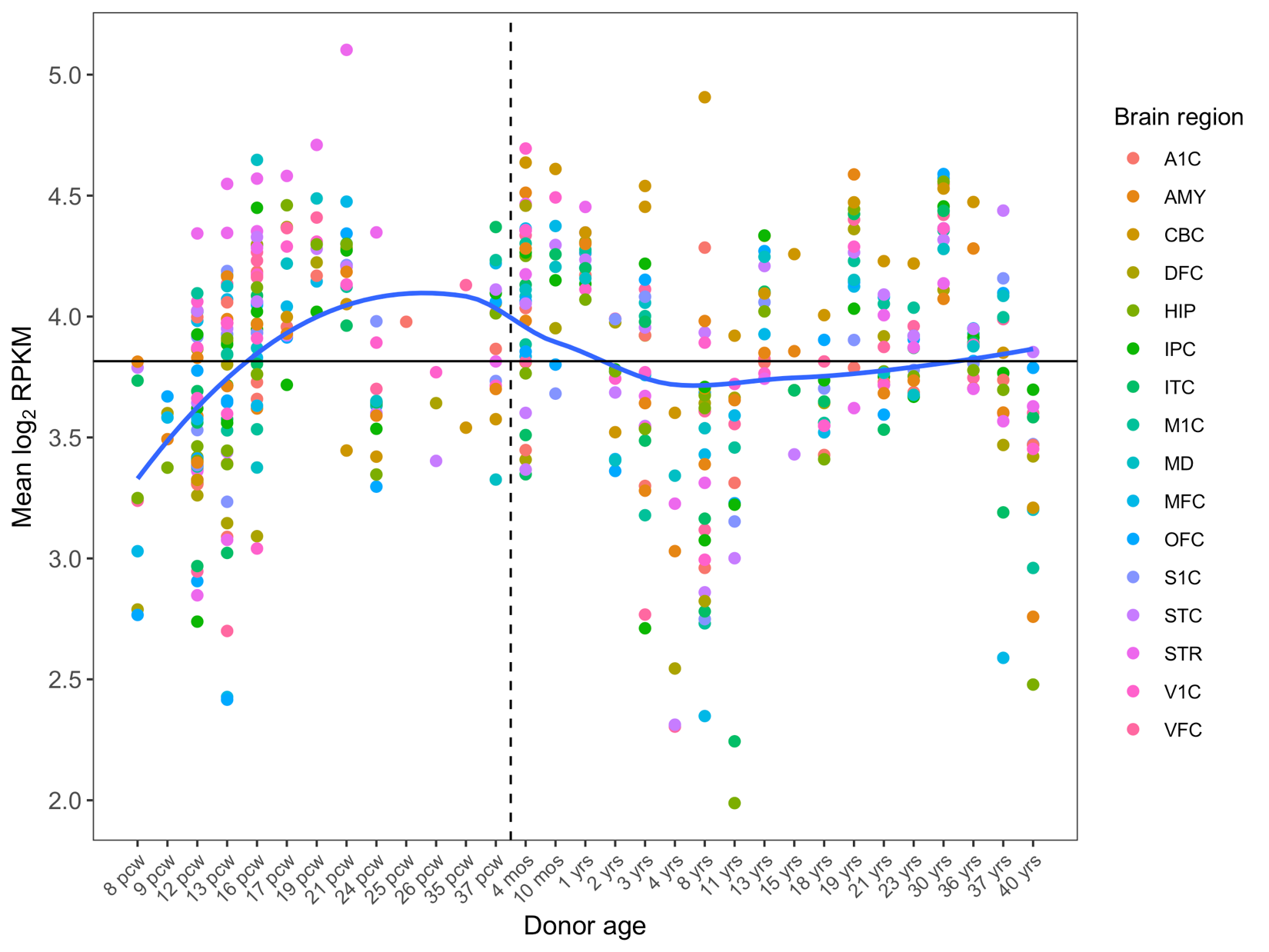


Supplementary Figure 4. Developmental trajectory of *TAOK2* expression in human *postmortem* brain samples across lifespan measured by RNA-sequencing. Donor ages ranged from 8 post-conception weeks (pcw) to 40 years old. The data were obtained from the BrainSpan: Atlas of the Developing Human Brain (<https://www.brainspan.org/>). The vertical dotted line denotes birth, and the solid horizontal line represents the average expression of *TAOK2* over all ages and brain regions. The blue LOESS regression line depicts a smoothed relationship between age and expression level. Data were limited to brain regions with a minimum of 10 donors. Expression levels of *TAOK2* were averaged over donors, and presented as mean log_2_ reads per kilobase per million mapped reads (RPKM).


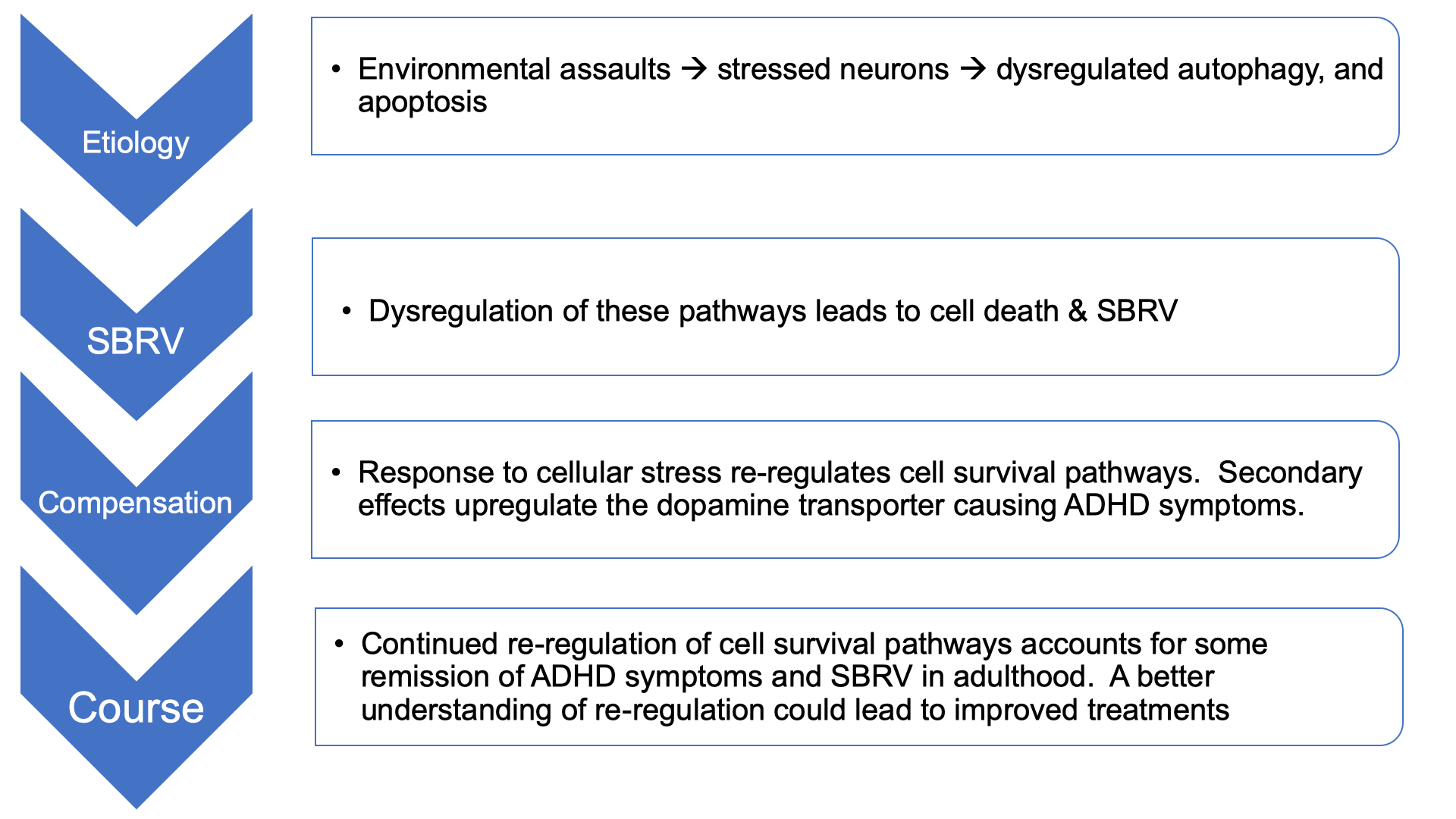


Supplementary Figure 5. The working model that integrates environmental exposures, regional gene set expression in the brain, and cellular stress into our selective brain region vulnerability (SBRV) hypothesis for attention-deficit/hyperactivity disorder (ADHD).

# References

1 Hoogman M, Bralten J, Hibar DP, Mennes M, Zwiers MP, Schweren LSJ *et al.* Subcortical brain volume differences in participants with attention deficit hyperactivity disorder in children and adults: a cross-sectional mega-analysis. *The Lancet Psychiatry* 2017. doi:10.1016/S2215-0366(17)30049-4.

2 Hoogman M, Muetzel R, Guimaraes JP, Shumskaya E, Mennes M, Zwiers MP *et al.* Brain Imaging of the Cortex in ADHD: A Coordinated Analysis of Large-Scale Clinical and Population-Based Samples. *Am J Psychiatry* 2019. doi:10.1176/appi.ajp.2019.18091033.

3 Shen EH, Overly CC, Jones AR. The Allen Human Brain Atlas: comprehensive gene expression mapping of the human brain. *Trends Neurosci* 2012; **35**: 711–714.
